# Profiles of Intimate Partner Violence Victimization: A Systematic Review

**DOI:** 10.1177/15248380221126183

**Published:** 2022-10-05

**Authors:** Maisie Hall, Emily Hill, Georgia Moreland, George K. Hales, Daniel Boduszek, Agata Debowska

**Affiliations:** 1 The University of Sheffield, UK; 2 University of Huddersfield, UK; 3 SWPS University of Social Sciences and Humanities, Poland

**Keywords:** intimate partner violence, latent class analysis, latent profile analysis, profiling research, systematic review

## Abstract

Person-centered approaches, such as latent class analysis (LCA) and latent profile analysis (LPA), aid the identification of subgroups within sample populations. These methods can identify the patterns of co-occurrence between different forms of intimate partner violence (IPV), providing valuable information for prevention and intervention efforts. The aim of this systematic review was to yield a summary and conduct a critical evaluation of the current research that utilizes LCA/LPA to investigate IPV victimization profiles. We provide an outline of 14 relevant studies, retrieved from searches conducted on PsycInfo, Scopus, and Eric databases. There was a large amount of variability in relation to the forms of IPV assessed, measures utilized, number of classes identified, and the sample populations recruited. However, broad similarities were revealed as there were some commonly identified classes, including the no/low violence class, the physical and psychological victimization class, and the multiple victimization class, yet the labels assigned to those classes differed across studies. A range of external criteria (risk factors and consequences) were also identified as being associated with class membership. We highlight the methodological features which may have impacted data collection and class enumeration, including the differences in sample population, the range of IPV indicators assessed, the time period from which IPV data were recorded, and whether data were collected regarding participants’ current or previous relationships. Marginalized populations were underrepresented, and psychological abuse was most inconsistently operationalized. Recommendations for future research are provided, including recommendations with regard to labeling the classes for greater consistency across studies.

Referring to behaviors perpetrated by a current or former romantic partner which cause physical, sexual, or psychological harm (World Health Organization [WHO], 2021), intimate partner violence (IPV) is regarded as a severe public health crisis (Trabold et al., 2020). IPV victimization is widespread, paying little regard to the gender, sexuality, status, or culture of the individual (Evans et al., 2020), and despite problems with underreporting (Chan, 2012), prevalence figures provided by official statistics remain high. Indeed, one in four women and one in ten men are subjected to any form of IPV victimization (Evans et al., 2020). This problem has been exacerbated by the COVID-19 lockdown restrictions implemented throughout many countries (Agüero, 2021; Lyons & Brewer, 2022), with forced cohabitation and restricted support services resulting in increasingly limited opportunities for victims to leave their abuser, and environmental stressors heightening the risk of IPV perpetration (Jarnecke & Flanagan, 2020). As for consequences, a wealth of research has highlighted the number of damaging and persistent sequelae associated with IPV victimization, including psychological, physical, and social impacts (e.g., Ansara & Hindin, 2011; Chisholm et al., 2017; Gupta et al., 2018; Iverson et al., 2017; Loxton et al., 2017; Simmons et al., 2018; Wood & Sommers, 2011).

The configurations of IPV experiences, however, differ substantially between individuals with regard to the severity, frequency, and type of abuse suffered, thus the introduction of Johnson’s (2008) typologies of IPV initiated a critical advancement in IPV literature. He proposed distinct categories of abuse characterized by variations in the use of control tactics, the context of the abuse, and the extent to which the perpetration of abuse is gender symmetric, highlighting the need for researchers to approach IPV as a multidimensional phenomenon and providing a conceptual framework which has since been developed by many subsequent studies (e.g., Carbone-López et al., 2006; Podaná, 2021). Upon considering the multifaceted nature of IPV, the unsuitability of traditional, variable-centered approaches for the statistical analysis of IPV data becomes increasingly apparent, given that such techniques focus solely on describing relationships between study variables and fail to consider possible differences in individual experiences (Howard & Hoffman, 2018). Consequently, variable-centered statistical approaches treat IPV as a unitary concept where each experience is deemed to exert an equal amount of physical or psychological impact, limiting the accuracy of the conclusions drawn from these studies and leading to questions surrounding the validity of results.

The ability for researchers to substantiate specific subgroups of IPV victims is essential for the progression of IPV research for several reasons. First, the prevalence of specific types or combinations of abuse can be compared across populations to discern individuals who may be most at risk of suffering particular abuse patterns. Furthermore, associated outcomes and specific consequences of distinct abuse patterns can be assessed to determine which configurations of abuse elicit the most severe ramifications, and enable researchers to link specific IPV typologies to specific outcomes. This will also help to improve both the specificity of intervention programs and the allocation of resources, as well as facilitate more thorough assessments of intervention efficacy and outcomes (Ali et al., 2016). Finally, identifying IPV typologies could enable researchers to identify specific etiological factors which lead to the experience of different abuse typologies, which, in turn, would contribute toward the development of better prevention techniques through targeting risk factors.

To reconcile the limitations generated by the use of variable-centered approaches, a shift in the statistical techniques used in the analysis of IPV data has been observed over recent years as researchers have turned toward using person-centered statistical approaches: specifically latent class analysis (LCA) and latent profile analysis (LPA).

## What are LCA and LPA?

LCA and LPA refer to person-centered statistical approaches which seek to uncover hidden (or latent) groups of homogeneous individuals within a population, deduced from responses to a number of categorical (in the case of LCA) or continuous (in LPA) predictor variables (Nylund-Gibson & Choi, 2018). Theoretical comparisons are commonly made between LCA/LPA and factor analysis, although the key difference is that factor analysis is used to group items, whereas LCA and LPA are used to group individuals—hence their classification as “person-centered” analyses (Nylund-Gibson & Choi, 2018).

Groups which are elucidated through LCA/LPA are finite and mutually exclusive, meaning that the final selection of classes should explain the patterns visible in the data in a way that shows how each member of the population belongs to only one class. The process of determining the optimal number of classes to satisfy these requirements is known as class enumeration (Nylund-Gibson & Choi, 2018), where multiple different models, each involving a different number of classes, are fitted to the data. It is recommended that researchers begin the process by fitting a model with only one class, before iteratively adding additional classes to assess which model provides the best fit and the most accurate description of the data. The fitting of additional classes should cease when no more empirical or theoretical evidence exists to support further classes, indicated through fit indices and considerations of parsimony and conceptual cohesiveness (Porcu & Giambona, 2017).

The indices used to assess model fit in LCA/LPA can be broadly divided into three categories: likelihood-based tests, goodness of fit indices, and entropy. First, the likelihood-based tests used in LCA/LPA—namely the Lo–Mendell–Rubin likelihood ratio test (Lo et al., 2001) and the bootstrapped likelihood ratio test (Arminger et al., 1999)—provide *p* values as an indication of whether the addition of each individual class results in a statistically significant improvement in the model fit. Thus, the observation of a non-significant *p* value (*p* > .05) can be interpreted as an indication that the model without the most recently added class offers the more parsimonious solution (Debowska et al., 2017). Conversely, goodness of fit indices—specifically Bayesian information criterion (BIC; Schwarz, 1978), Akaike’s information criterion (Akaike, 1974) sample-size adjusted BIC (_SSA_BIC; Sclove, 1987) the G^2^-test (Agresti, 2003), and Pearson’s χ^2^ statistic (Pearson, 1900)—are used to compare the goodness of fit across the different models being tested, where a lower value indicates a better model fit. It should be noted that while better assessments of model fit are achieved through considering as many fit indices as possible, BIC values are thought to offer the most reliable indication of model fit and should therefore be given more emphasis if a final class solution is not clear (Nylund et al., 2007).

Finally, entropy values (Ramaswamy et al., 1993) indicate the degree of distinction between classes, where higher values represent greater separation. Entropy values are an aggregate of posterior class probabilities (Roesch et al., 2010)—values derived from a combination of participant responses, the number of classes included in the model, and the proportion of participants allocated to each class—and such values indicate how well a model categorizes individuals into their most suitable class (Nylund-Gibson & Choi, 2018; see also Kaplan & Keller, 2011). Possible entropy values range from 0 to 1, and it is often recommended that researchers implement a cutoff value of .80 indicating that individuals are allocated to the correct group 80% of the time (Clark & Muthén, 2009).

In addition to assessing model fit, the conceptual coherence of classes can be assessed through the interpretation of model parameters: latent class probabilities (LCPs) and conditional response probabilities (CRPs) when conducting an LCA, or LCP and conditional response means (CRMs) in the case of LPA. LCP values represent the most probable latent class membership for each individual (Debowska et al., 2017; Lanza & Collins, 2008); CRP figures denote the probability of each IPV indicator included in the study being present within each class; and CRM values signify the mean value of each observed indicator within each latent group. Comparison of CRPs can be used to inform the process of naming the retrieved classes (Vermunt & Magidson, 2004).

Finally, once the optimal class solution has been selected, the association between class membership and theoretically driven covariates can be assessed through further statistical tests. Researchers are encouraged to examine such associations given that differential associations between the separate latent classes and specific outcome variables can reinforce the external validity of the derived classes (Petersen et al., 2019).

## The Current Study

Given the relatively novel application of LCA/LPA to IPV research, the current paper aims to provide a systematic summary and critical review of all studies which have applied LCA/LPA techniques to the analysis of IPV data. Collating these findings will enable the identification and comparison of all latent classes and facilitate a discussion around any inconsistencies which are found between classes retrieved by different studies. This will also enable the assessment of which latent classes have been replicated and substantiated by other research, and likewise identify the areas of knowledge which require further development. Recommendations for future research will also be provided.

## Method

### Procedure

**Figure 1.**
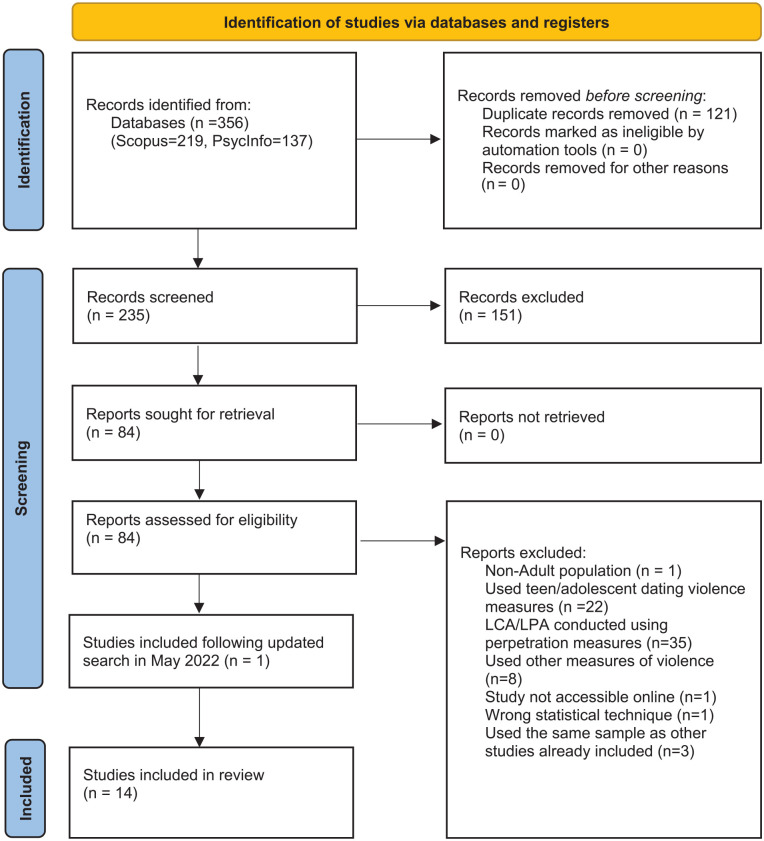
PRISMA flow diagram showing the process of identifying studies for the review.

This review was carried out according to Siddaway et al.’s (2019) recommendations for conducting psychological systematic reviews. Searches were conducted on PsycInfo and Scopus databases in March 2021 using combinations of the following search terms: *intimate partner violence, IPV, domestic violence, domestic abuse, dating violence*, AND *latent class analysis, LCA, latent profile analysis, LPA, latent class, latent profile*. The results generated across both databases produced 356 papers. An additional, subsequent search was then conducted via Eric to ensure that no relevant papers had been missed. The papers were then exported to the reference management software Mendeley Desktop Version 1.19.8 to remove duplicate papers (*N* *=* 121), leaving a total of 235 studies. The title, abstract, and method sections of all remaining papers were then read by three researchers to establish inter-rater reliability when determining which papers were applicable to the current review, and any irrelevant papers were discarded. One additional study was identified through the bibliography of the reviewed papers, resulting in a final number of 13 papers from across 10 different journals. A supplementary search of PsycInfo and Scopus databases was conducted in May 2022 to find any additional studies published between the original search and the completion of review (March 2021–February 2022). This search yielded 21 papers, of which one fit inclusion criteria. Consequently, 14 studies were included in the review. For a PRISMA diagram detailing this process, see Figure 1.

### Selection Process

To be included in the current review, papers were required to meet the following criteria:

1. LCA or LPA was utilized for the retrieval of IPV classes or profiles.
2. A measure of IPV was used (as opposed to a measure of Teen Dating Violence or other general violence).
3. The LCA/LPA included indicators of IPV victimization only (as opposed to a mixture of IPV and non-IPV victimization experiences or a mixture of IPV victimization and perpetration).
4. The sample was limited to adult populations only. If populations spanned adolescence and adulthood, the study was included providing that a measure of IPV was used.
5. No restrictions were implemented regarding the types of IPV being assessed.
6. The paper was written in English.
7. The paper was published in a peer-reviewed journal.

### Data Extraction and Analysis

Data were extracted from each study regarding the paper’s author(s), date of publication, sample population, method of data collection, types of IPV measured (and measures used), specified period of IPV, the number and names of IPV classes retrieved, the entropy value of the chosen class solution, and any external criteria included in the study to determine the external validity of the derived classes. This information is provided in Table 1, and full results are presented in a narrative review below. Classes which are labeled ambiguously have been described in the narrative review. Finally, it is worth noting that although both LCA and LPA were included in the review’s search terms, all 14 studies employed LCA due to their use of categorical predictors, and no studies using LPA were retrieved. Those 14 studies reported the results of 17 LCAs, with three studies analyzing data separately for women and men. Due to the heterogeneity of selected studies, quantitative analysis of data was not feasible. Therefore, the results are presented as a narrative review.

**Table 1.** Summary of Method and Results of Each Paper Included in Review (*N* = 14).

Authors(s) and Year of Publication	Study Population and Method of Data Collection	Types of IPV and Time Period Measured (Measure Used)	IPV Groups Retrieved and Percentage of Participants	Entropy Value	External Criteria
Ansara and Hindin (2010)	15,416 adults (8,360 women and 7,056 men; aged >15 years; secondary data drawn from the 2004 Canadian GSS—computer-assisted telephone interview)	Emotional and financial abuse at any point (7 binary items); Physical and sexual violence in the previous 5 years (a modified version of the CTS; Straus, 1979).	6 classes for women: no violence or abuse (85.1%); jealousy, verbal abuse (8.1%); physical aggression (2.6%); severe violence, control, verbal abuse (1.8%); physical aggression, control, verbal abuse (1.3%); control, verbal abuse (1.1%) 4 classes for men: no violence or abuse (90.3%); jealousy, verbal abuse (5.3%); physical aggression (2.8%); moderate violence, control, verbal abuse (1.5%)	—	Violence-related characteristics, relationship status—current vs. ex-partner (risk factor)
Cale et al. (2017)	293 female undergraduate university students in Australia and New Zealand (*M* = 22.8 years; secondary data drawn from the IDVS—self-report)	Minor and severe psychological and physical aggression and injury, and sexual abuse (insisting, threats and force) experienced at any point (items derived from the CTS2; Straus et al., 1996).	3 classes: low-level IPV (53%); moderate-level IPV (35%); and high-level IPV (12%)	.76	Child abuse (risk factor), depression (consequence)
Carbone-López et al. (2006)	11,858 U.S. adults (5,867 men and 5,991 women; aged <65 years; secondary data drawn from the NVAWS—telephone interview)	Physical violence, sexual abuse, stalking (items that resembled the CTS; Straus, 1979)	4 classes for men: no IPV (91.7%); interpersonal conflict violence (3.4%); physical aggression (3.3%); and systematic abuse (1.6%) 4 classes for women: no IPV (77%); interpersonal conflict violence class (11.3%); physical aggression (8.2%); and systematic abuse (3.5%)	—	Physical health, psychological distress, substance abuse (consequences)
Clark, Cheong, et al. (2019)	1,440 married women from Nepal (aged 18–49 years; drawn from the What Works to Prevent Violence Against Women and Girls consortium—self-report survey)	Psychological abuse, physical violence, sexual abuse during the previous 12 months (What Works to Prevent Violence Against Women and Girls consortium; Clark, McGhee, et al., 2019)	4 classes: low exposure (77.36%); sexual violence (9.03%); moderate violence (6.6%); and systematic violence (7.01%)	.93	Depressive symptoms (consequence)
Gupta et al. (2018)	947 low-income women using community health clinics in Mexico City (18–44 years, *M* age = 30 years; data drawn from a completed RCT—self-report)	Physical violence, sexual violence, injuries during the past year (items from the WHO [2005] Multi-Country study on Women’s Health and Domestic Violence)	4 classes: low physical and sexual violence and low injuries (39.1%); high sexual and low physical violence (9.6%); high physical and low sexual violence and injuries (36.5%); and high physical and sexual violence and low injuries (14.8%)	.80	Work disruption (consequence)
Lagdon et al. (2022)		Physical abuse, sexual abuse, harassment, emotional denigration, emotional restrictive engulfment, emotional dominance/intimidation in the past 12 months (24 items from the 30-item CAS; Hegarty et al., 1999)	3 classes for men: low or no IPV (48.37%); male physical abuse/emotional denigration victimization (34.24%); and male physical/emotional abuse & harassment victimization (17.39%) 3 classes for women: low or no IPV (56.24%); female physical abuse/emotional denigration victimization (27.42%); and female IPV polyvictimization (16.34%)	.88	PTSD, depression, anxiety, alcohol use (consequences)
Leyton (2020)	2,256 women with a child aged 3–4 in Honduras (aged 15–49 years, *M* = 29.8 years; secondary data obtained from the 2011 to 2012 Honduras DHS—interview)	Controlling behaviors, emotional abuse, physical violence, and sexual abuse during the current or most recent relationship, and physical and sexual abuse during any previous relationship (modified version of the CTS; Straus, 1996).	5 classes: no violence (64.7%); physical and sexual violence by an ex-partner (6.8%); current emotional violence (14.9%); current controlling, emotional, and physical violence (8.1%); and past controlling, emotional, and physical violence (5.4%)	.90	ECD outcomes among victim’s children (consequence)
Liu et al. (2018)	610 U.S. parent–adolescent dyads (58.2% female parents; secondary data drawn from the National STRiV—self-report survey)	Verbal abuse and physical violence in the past year (items adopted from various sources).	3 classes: low IPV (81.8%); verbal abuse (14.1%); and high IPV (4.2%)	.95	Adolescent relationship abuse among victim’s children (consequence)
Lysova and Dim (2020)	52,400 Canadian men (aged >15 years; secondary data drawn from the 2009 (*N* = 19,400) and 2014 (*N* = 33,000) editions of the Canadian GSS on Victimization—computer-assisted telephone interviews)	Physical violence, psychological abuse, sexual IPV, and experiences of injuries within the past 5 years (a modified version of the CTS; Straus et al., 1996)	4 classes: milder physical violence only (57.3%); jealousy and milder physical violence (19.2%); moderate physical violence (13.8%); and severe physical and psychological violence (9.7%)	—	Help-seeking behaviors (consequence)
McNaughton Reyes et al. (2019)	561 South African women who were newly diagnosed as HIV-positive during pregnancy (aged >18, *M* = 26.4 years; data drawn from HIV counseling RCT—face-to-face interview)	Moderate and severe psychological abuse, moderate and severe physical abuse, sexual IPV, and male controlling behavior during or prior to pregnancy by their current partner (a modified version of the WHO [2005] Multi-Country study on Women’s Health and Domestic Violence)	3 classes: non-victims (74%); moderate IPV (20%); and multiform severe controlling IPV (5%)	.77	Postpartum unsafe sexual activity (consequence)
Mcnaughton Reyes et al. (2021)	1,480 pregnant women in South Africa (aged 18–45 years, *M* = 26 years; data drawn from HIV counseling RCT—computer-assisted personal interviews)	Psychological abuse, physical violence, sexual abuse, and male controlling behavior at any point in the current relationship (a modified version of the WHO [2005] Multi-Country study on Women’s Health and Domestic Violence)	3 classes: non-victims (72%); moderate IPV (24%); and multiform severe controlling (4%)	.76	Emotional distress during and post-pregnancy (consequence)
Podaná (2021)	30,675 EU women (aged 18–74 years; secondary data drawn from an EU survey on violence against women—face-to-face interviews)	Psychological abuse, physical violence, and sexual violence at any point in their current relationship (a 30-item self-constructed scale)	5 classes: no violence (83.7%); intimate terrorism (1.5%); high coercive control (2%); situational couple violence (3.8%); and situational psychological abuse (9.0%)	—	Women’s characteristics linked to vulnerability of abuse or dependency on their partner; women’s history of abuse; risk factors related to male perpetration and violent behavior outside of the family; cross-country variation (risk factors)
Whitton et al. (2019)	352 FAB SGM youth (aged 16–32 years)—interview	Minor and severe psychological IPV, coercive control, minor and severe physical IPV, injury, sexual IPV, cyber dating abuse, SGM-specific IPV in the past 6 months (the SGM-CTS2; Dyar et al., 2021).	3 classes: no/low IPV (52.5%); psychological IPV (32.1%); and high IPV (15.3%)	.73	Sexual and gender identity, race/ethnicity, partner gender identity, age (risk factors)
Willie et al. (2020)	593 low-income women seeking support from a community health clinic in Mexico City—clinic-based data self-report	Physical IPV, sexual IPV, and reproductive coercion during the past year (self-constructed questions).	3 classes: high physical/high sexual IPV and high reproductive coercion (16.4%); low physical/low sexual IPV and low reproductive coercion (69.8%); and high physical/low sexual IPV and low reproductive coercion (13.8%)	—	—

*Note.* Cells are left blank if information was not provided in the study.

CAS = Composite Abuse Scale; CTS = Conflict Tactics Scale; CTS2 = Revised Conflict Tactics Scale; DHS = Demographic Health Survey; ECD = early childhood development; EU: European Union; FAB SGM = female-assigned-at-birth sexual and gender minority; GSS = General Social Survey; IDVS = International Dating Violence Study; IPV = intimate partner violence; NVAWS = National Violence Against Women Survey; PTSD = post-traumatic stress disorder; RCT = randomized control trial; SGM-CTS2 = sexual and gender minority CTS2.

## Results

Across the 14 reviewed studies, a three-class model was identified as the optimal solution eight times, a four-class models—six times, a five-class model—twice, and a six-class model—once. As for types of IPV, all studies included measures of physical IPV, and 13 studies included measures of sexual IPV. Most variation was found in the measures of psychological abuse. Specifically, although 12 studies assessed this form of IPV, some studies differentiated between psychological IPV and more specific components, such as coercive control, controlling behaviors, verbal abuse, jealousy, and financial abuse. As for methods of assessment, five studies used a modified version of the Conflict Tactics Scale (Straus, 1979) or the Revised Conflict Tactics Scale (Straus et al., 1996) and three studies used items from the WHO (2005) Multi-Country study in Women’s Health and Domestic Violence. The remaining studies used items derived from other sources or self-constructed scales. In all, 13 studies assessed external criteria associated with IPV victimization. These were both risk factors which may lead to certain forms of IPV victimization (four studies) and possible consequences of IPV victimization (10 studies). Finally, entropy values for the best LCA solutions were reported in nine studies. In four analyses, those values were below the recommended cutoff point of .80. More detailed description of study properties is found in Table 1.

Results below are grouped into five subsections based upon the nature of IPV groups retrieved: (1) no/low IPV groups, (2) single IPV groups, (3) physical and psychological IPV groups, (4) multiple IPV groups—physical, psychological, and sexual IPV, and (5) other multiple IPV groups.

### No/Low IPV Groups

Classes labeled by researchers as no/low IPV groups were retrieved in 13 of the reviewed studies and across 16 analyses (Ansara & Hindin, 2010; Cale et al., 2017; Carbone-López et al., 2006; Clark, Cheong, et al., 2019; Gupta et al., 2018; Lagdon et al., 2022; Leyton, 2020; Liu et al., 2018; Podaná, 2021; McNaughton Reyes et al., 2019, 2021; Whitton et al., 2019; Willie et al., 2020). Membership in those classes ranged from 39.1% (Gupta et al., 2018) to 91.7% (Carbone-López et al., 2006), with a mean of 69.79% (SD = 15.91%) across all 16 analyses.

The lowest class membership in the no/low abuse classes (at least 1 SD below the mean, i.e., below 53.88%) was reported by Gupta et al. (2018), Lagdon et al. (2022), Whitton et al. (2019), and Cale et al. (2017). According to Gupta et al. (2018), merely 39.1% (1.93 SD below *M*) of low-income women using community health clinics in Mexico City did not experience or experienced low levels of IPV. In Whitton et al.’s (2019) research among sexual and gender minority (SGM) young people who were assigned female at birth (FAB), including sexual minority women, transgender men, and non-binary FAB individuals, no/low IPV group membership amounted to 52.5% (1.09 SD below *M*). Both studies focused on marginalized populations, which are known to have an increased risk of IPV victimization. Furthermore, in Cale et al.’s (2017) study among female undergraduate university students (*M*_age_ = 22.8 years) from Australia and New Zealand only 53% (1.06 SD below *M*) of participants were classified in the low-level IPV group. Although this finding may seem unexpected, prior research indicates that females aged between 20 and 24 years are at the greatest risk of non-fatal IPV (Catalano, 2006; Office for National Statistics, 2018). In another study among university students, Lagdon et al. (2022) found that 48.37% (1.35 SD below M) of men in the sample experienced no or low IPV. The researchers conducted LCA on six IPV indicators including four indicators of psychological IPV. In comparison, 56.24% of female students in the same study were classed in low or no IPV group. However, it should be noted that female students in the low or no IPV group had higher risk of reporting five out of six assessed IPV forms, compared with their male counterparts.

The highest class membership in the no/low abuse classes (at least 1 SD above the mean, i.e., above 85.7%) was reported by Carbone-López et al. (2006)—91.7% among men (1.38 SD above *M*)—and Ansara and Hindin (2010)—90.3% among men (1.29 SD above *M*). Both studies used North American samples. These high percentages could be partly explained by the data collection methods. More specifically, both studies utilized data from national surveys, where participants had been interviewed about abuse by current or ex-partner via computer-assisted telephone interviewing. Therefore, participants with a current partner could be in close proximity to their partner while answering the survey questions, which could have affected their honesty. For example, they could have decided not to report IPV perpetrated by their current partner in an attempt to protect them, or may have felt unable to disclose their situation out of fear of their abuser. Corroborating this point may be information provided in Ansara and Hindin’s (2010) study, which assessed the prevalence of IPV classes for those reporting about a current versus an ex-partner. It was found that 94.1% of women and 95.2% of men reporting on violence by current partner, compared with 51.4% of women and 66.2% of men reporting on violence by ex-partner, were classified in the no violence or abuse class. Alternatively, another possibility is that individuals who were in abusive relationships in the past left these relationships to find non-abusive partners. In addition, Ansara and Hindin noted that participation in the survey did not require informed consent. Therefore, participants could have been worried about confidentiality and anonymity of their responses, yet another factor affecting reliability. It is also worthy of note that Ansara and Hindin (2010) and Carbone-López et al. (2006) performed separate analyses among women and men. Both studies demonstrated that more men than women are IPV free, substantiating statistical evidence from prevalence research indicating that women are more likely to experience IPV (Tjaden & Thoennes, 2000).

Finally, although there were 13 papers/16 analyses in this review that retrieved classes that were labeled as no/low IPV, there were only five papers/six analyses in which this class recorded less than 5% risk of reporting IPV behaviors (Ansara & Hindin, 2010—one analysis with men; Carbone-López et al., 2006—both analyses; Clark, Cheong, et al., 2019; Lagdon et al., 2022—one analysis with men; McNaughton Reyes et al., 2019). Of note, Lagdon et al. (2022), who conducted separate analyses for women and men, reported a “low or no IPV” class for both genders; however, only men in this class had less than 5% risk of reporting all assessed IPV behaviors. Women in the same class, in turn, had a 14% risk of reporting emotional denigration. It appears that some researchers label a class as “no/low abuse” because participants in the group experience less IPV relative to the remaining participants in that sample, but these groups may not be comparable across studies. In yet another example, in Gupta et al.’s (2018) “low physical and sexual violence and low injuries” group with 39.1% participants (as discussed above), the risk of having experienced physical abuse in the form of being pushed or shoved amounted to approximately 94%. The risk of having bruises was 35%, which may indicate that the physical abuse experienced was not minor. Cale et al.’s (2017) “low-level IPV” group, in turn, recorded 52% risk of emotional abuse and 12% risk of sexual abuse. This further highlights the ubiquity of IPV and the plight of some communities—a piece of information that could be lost behind the unfortunate class labels. To better understand the patterns of IPV across samples and communities, it appears crucial that group labels are reflective of the risks associated with a particular class membership, as opposed to being complementary to the remaining classes retrieved in the same analysis. It appears that such an approach was taken by the only study in this review that did not identify a no/low IPV class. Specifically, Lysova and Dim (2020) had a class labeled “milder physical violence only” with 43% risk of physical abuse, 0% risk of sexual assault, and 11% risk of emotional abuse. To systemize research in the area, it may be helpful if the research community agreed on the level of risk above which a class should not be labeled “no/low IPV.”

### Single IPV Groups

Here, we report on LCA classes with an increased risk (>10%) of a single type of IPV and very low risk (≤5%) of experiencing remaining IPV types included in the analysis. Four classes fulfilling these criteria were found in three different studies (Carbone-López et al., 2006; Liu et al., 2018; Whitton et al., 2019). The low number of such groups demonstrates that single IPV forms are not usually experienced in isolation. Carbone-López et al. (2006) found a physical aggression group among men (3.3% class membership). Physical aggression was measured with seven items and risk of experiencing those behaviors by men in the group ranged between 4.7% (being choked or beaten up) and 91.5% (being pushed). A class with the same label was also reported for women (8.2% class membership). However, women in this class, in addition to having a high risk of physical aggression, also recorded an increased risk of sexual assault (11.5%) and stalking (14.5%). This is yet another example of how striving for consistency in class labeling across analyses within one study can obscure the nature of IPV experienced by different groups of individuals. Furthermore, there were two instances where single IPV groups were presented as no/low IPV groups (Liu et al., 2018; Whitton et al., 2019), even though the risk of experiencing verbal abuse (Liu et al., 2018) and minor psychological abuse (Whitton et al., 2019) by members of those classes was 30% and 35% respectively.

### Physical and Psychological IPV Groups

In this subsection, we have included LCA classes with an increased risk (>10%) of at least one form of physical and psychological IPV and very low risk (≤5%) of experiencing remaining IPV types included in the analysis. Definitions of psychological abuse differ across the reviewed studies. Therefore, for the purpose of the current study, psychological IPV is considered to include any type of emotional abuse as well as coercive and controlling behavior. “Emotional abuse involves behaviors intended to generate emotional harm or threat of harm, such as belittling, humiliating, threatening or intimidating the victim, whereas controlling behavior entails monitoring partner’s behaviors or isolating them by limiting actions, such as forbidding them to leave the house, restricting contact with other people, or continually insisting on knowing the victim’s whereabouts” (Martín-Fernández et al., 2019, p. 2). Using criteria listed above, we identified eight physical and psychological IPV classes across four studies (Ansara & Hindin, 2010; Leyton, 2020; Liu et al., 2018; Lysova & Dim, 2020).

Ansara and Hindin (2010) as well as Lysova and Dim (2020) each found three classes characterized by increased physical and psychological IPV. In both studies, Canadian General Social Survey (GSS) on victimization data were utilized but from different waves. In Ansara and Hindin’s (2010) research, two of those classes were retrieved for men and one for women. Among women, the class was labeled “control, verbal abuse” (1.1% class membership), with item-response probabilities for psychological abuse of up to 100% (“puts you down/calls you names”) and for physical abuse of up to 78% (“damages/destroys possessions/property”). Among men, the groups were labeled “physical aggression” (2.8% class membership) and “moderate violence, control, verbal abuse” (1.5% class membership). The main difference between the two classes was that men in the “moderate violence, control, verbal abuse” class recorded increased risk (up to 93%) of experiencing all six psychological IPV behaviors compared with increased risk (up to 57%) of experiencing three psychological IPV behaviors in the “physical aggression” class. Despite the class labels, the risk of having experienced physical abuse was also higher among men in the “moderate violence, control, verbal abuse” class, compared with the “physical aggression” class. Noteworthy, all three classes are characterized by small membership (<5%), which can be considered a statistical anomaly (Hipp & Bauer, 2006). In such cases, the stability of classes should be assessed across studies.

Lysova and Dim (2020) used data collected in the 2009 and 2014 editions of the Canadian GSS to investigate IPV occurrences of Canadian men (*N* = 52,000) who were married or in a common-law relationship between 2004 and 2014. Experiences of physical, psychological, sexual IPV, and injuries were analyzed. Three out of four latent classes were characterized by increased physical and psychological IPV, namely, “milder physical violence only” (57.3% class membership), “jealousy and milder physical violence” (19.2%), and “moderate physical violence” (13.8%). Risk of different forms of psychological abuse was the highest for the “jealousy and milder physical violence” group (up to 70%). The “moderate physical violence” class, in turn, recorded the highest risk of having experienced physical abuse (up to 80%).

Liu et al. (2018) focused on parent–adolescent dyads in the United States to assess whether parental IPV experiences impacted upon their adolescent children’s dating behavior. Three classes were identified, with “high IPV” class (4.2%) being characterized by increased physical and psychological IPV. It was also found that parents who were married were more likely to be in the high IPV group compared to those in dating relationships, which is a contradictory finding to many previous studies who cite marriage as a protective factor from IPV (for a review, see Yakubovich et al., 2018). However, congruent with previous findings, cohabiting relationships led to more risk (Castro et al., 2017; Manning et al., 2018), and parents were more likely to belong to the high IPV class if their income was categorized as being below the poverty line (Matjasko et al., 2013). IPV indicators were restricted, however, to verbal abuse and physical violence during the past year, making Liu et al.’s (2018) study the least comprehensive in terms of IPV measures. To gain a better understanding of how different IPV forms co-occur with one another, future research should aim to measure more diverse IPV types where possible.

Leyton (2020) examined IPV experiences among Honduran mothers aged 15–49 years old with a child aged 3–4 years. Physical, sexual, emotional IPV, and controlling behaviors perpetrated by a current (or most recent) partner, and physical and sexual experiences from an ex-partner were measured. LCA yielded a five-class solution. One of the groups, the “current emotional violence” class (14.9% class membership) was characterized by increased risk of emotional abuse, controlling behavior, and physical abuse. Unlike studies utilizing external criteria of participant’s childhood history (Cale et al., 2017; Podaná, 2021), Leyton (2020) examined the consequence of a mother’s experience of IPV on their children’s development. Results indicated significant differences between all the groups compared, which strengthens the validity of the five-class model identified and casts a light on the domino effect of a parent’s IPV experience on their children. As for the “current emotional violence” class, children of women in this class had lower odds of being developmentally on track in the socioemotional domain, compared to children of women in the “no violence” class.

### Multiple IPV Groups—Physical, Psychological, and Sexual IPV

Here, we have included LCA classes with an increased risk (>10%) of at least one form of physical, psychological, and sexual IPV and very low risk (≤5%) of experiencing any other IPV types (e.g., stalking, cyber abuse) included in the analysis. Based on these criteria, we found 20 physical, psychological, and sexual IPV classes across 10 studies (Ansara & Hindin, 2010; Cale et al., 2017; Clark, Cheong et al., 2019; Lagdon et al., 2022; Leyton, 2020; Lysova & Dim, 2020; McNaughton Reyes et al., 2019, 2021; Podaná, 2021; Willie et al., 2020). Three of those studies found three multiple IPV groups with varying levels of risk of experiencing different IPV forms within one analysis (Clark, Cheong et al., 2019; Leyton, 2020; Willie et al., 2020). Membership in those classes ranged from 1.3% (Ansara & Hindin, 2010) to 69.8% (Willie et al., 2020), with a mean of 12.78% (SD = 15.79%). This finding indicates that multiple IPV groups are commonly found across societies and IPV forms frequently co-occur. Although five of those classes had a class membership lower than 5% (Ansara & Hindin, 2010; McNaughton Reyes et al., 2021; Podaná, 2021), the commonness of the class across societies indicates that it is not a statistical anomaly.

The highest class membership in the multiple IPV classes (at least 1 SD above the mean, i.e., above 28.57%) was identified for two groups in two studies (Cale et al., 2017; Willie et al., 2020). These studies will be discussed in greater detail. First, Cale et al. (2017) measured minor and severe forms of psychological, physical, and sexual IPV incidents in a population of female undergraduate university students in Australia and New Zealand, given that being young and in a relationship but unmarried constitute risk factors for IPV victimization (Abramsky et al., 2011; Taşkale & Soygüt, 2017). Participants were asked to report experiences of IPV from across their whole lifetime. In the analysis, Cale et al. (2017) found a multiple IPV class which was labeled “moderate-level IPV” (35% class membership; 1.41 SD above *M*). Women in this group had a 100% risk of emotional abuse, 42% risk of physical abuse, and 50% of sexual assault. Interestingly, another similar class in this analysis, the “high-level IPV” class, with 94% of emotional abuse, 95% risk of physical abuse, and 64% of sexual assault, recorded a 12% class membership. This demonstrates that as many as 47% of young women experienced multiple victimizations. Participants from New Zealand were over-represented in the “moderate-level IPV” and “high-level IPV” groups. Members of the “high-level IPV” were more likely to report child neglect and violence victimization history as well as current depressive symptoms, compared with the “moderate-level IPV” and “low-level IPV” classes. Participants in the “moderate-level IPV” class, in turn, had a higher child neglect history score and current depressive symptoms score compared with the “low-level IPV.” This finding indicates that the two multiple IPV groups can be distinguished based on the presence and intensity of risk factors as well as the intensity of associated consequences—a crucial piece of information for targeted IPV intervention efforts.

Willie et al. (2020) included reproductive coercion as an indicator of IPV—referring to behavior which hinders women’s choices regarding fertility and contraceptive use—alongside physical and sexual IPV, which is unique to all other studies included in this review. For the purpose of the current study, we conceptualized reproductive coercion as a form of psychological IPV. The researchers found three multiple IPV classes, with the highest membership in the “low physical/low sexual IPV and low reproductive coercion” class (69.8% class membership; 3.61 SD above *M*). Although this class recorded 14% risk of reproductive coercion, 91% risk of physical abuse, and 15% risk of sexual abuse, the class label selected by the researchers suggests that members of this group experienced low levels of victimization. Indeed, these are “low levels” of IPV relative to other classes in the analysis, however, not in relation to classes retrieved in other studies—a labeling issue discussed above, which may obscure our understanding of IPV co-occurrence. Noteworthy, the remaining two groups retrieved in Willie et al.’s study also fulfil the above-listed criteria for multiple IPV classification: the “high physical/high sexual IPV and high reproductive coercion” (16.4% class membership) and “high physical/low sexual IPV, and low reproductive coercion” (13.8% class membership) classes. Therefore, *all* participants in this study experienced multiple victimizations, with the lowest risk recorded for sexual assault (15%) in the “low physical/low sexual IPV and low reproductive coercion” class. However, given that participants were recruited from a clinical sample, these results are not generalizable to other, non-clinical populations.

### Other Multiple IPV Groups

Four groups in two studies (Carbone-López et al., 2006; Whitton et al., 2019) were classified as other multiple IPV groups. These are groups characterized by an increased risk (>10%) of at least three types of IPV, but other than the combination of physical, psychological, and sexual IPV. Membership in those classes ranged from 8.2% (Carbone-López et al., 2006) to 32.1% (Whitton et al., 2019), with a mean of 14.78% (SD = 12.53%).

In the earliest study to apply LCA to IPV research, Carbone-López et al. (2006) investigated IPV experiences of U.S. men and women during both their current and previous marriages or cohabiting relationships. Physical abuse, sexual abuse, and stalking were assessed—making this the only study to include stalking as an IPV indicator. Threats of violence and weapon use were also assessed but were included as a measure of physical abuse. Carbone-López et al.’s analyses yielded a four-class solution for both male and female samples. Two female classes labeled “physical aggression” (8.2% class membership) and “systematic abuse” (3.5% class membership) recorded increased risk of all forms of measured IPV forms, with the “systematic abuse” class being characterized by higher risk of all IPV forms than the “physical aggression” class. The main difference between the two classes, therefore, was quantitative rather than qualitative. Associations were tested between latent class membership and physical health, mental health, and substance abuse. Compared with females in the “no IPV” class, their counterparts in the “physical aggression” class had elevated odds of injury, illness disability, having had a miscarriage, serious depression, mental health disability, as well as greater odds of using tranquilizers, sleeping pills, sedatives, antidepressants, prescription pain pills, and recreational drugs. Associations between systematic abuse and adverse consequences were the strongest. Specifically, women in the “systematic abuse” class, compared with women in the “no IPV” class, had twice the odds of having a miscarriage, using prescription pain pills, and drinking alcohol every day; two and a half times the increased odds of self-perceived poor health as well as using tranquilizers, sleeping pills, or sedatives; thrice the increased odds of an injury disability, serious depression, and using antidepressants; and four times the increased odds of a mental health disability.

Finally, Whitton et al. (2019), in a study among SGM young people who were assigned FAB, assessed a wide range of IPV indicators, including minor and severe psychological IPV, minor and severe physical IPV, sexual IPV, injury, and coercive control. This was also the only study to include measures of SGM-specific IPV and cyber dating abuse experiences. Three latent classes were identified, two of which represent other multiple IPV groups: “psychological IPV” (32.1% class membership) and “high IPV” (15.3% class membership). Similar to the Carbone-López et al.’s (2006) study, the two classes differed from each other mainly quantitatively. Using the “no/low IPV” as the reference group, the researchers found increased odds of Black and Latinx participants belonging in the “psychological IPV” than White participants. In addition, Black participants had four times the increased odds of being grouped into the “high IPV” class compared to White participants.

## Discussion

It is widely acknowledged that patterns of IPV differ between victims, and this premise is further substantiated by the variation in latent classes outlined in the current review. Across the 14 reviewed studies, a three-class model was identified as the optimal solution eight times, a four-class model—six times, a five-class model—twice, and a six-class model—once. Classes labeled by researchers as no/low IPV groups were retrieved in 13 of the reviewed studies and across 16 analyses. Belonging in the no/low IPV classes was associated with the least risk factors and adverse consequences. Furthermore, there were 20 multiple IPV classes characterized by an increased risk of physical, psychological, and sexual IPV victimization as well as four classes characterized by an increased risk of at least three types of IPV but other than the combination of physical, psychological, and sexual IPV victimization. Multiple IPV classes were predominantly the least prevalent and were commonly associated with increased risk factors and most adverse consequences. In addition, we identified eight classes characterized by an increased risk of physical and psychological IPV victimization and four classes characterized by an increased risk of a single abuse type.

Methodological variations across studies, including the use of different sample populations, rendered the results of different studies difficult to compare. IPV research predominantly focuses on female victims in heterosexual relationships, despite the emergence of research which suggests a more equal prevalence of IPV victimization across both genders (Breiding, 2015), and a heightened prevalence among bisexual individuals (Turell et al., 2018). In the current review, however, all three studies which conducted an LCA for each gender (Ansara & Hindin, 2010; Carbone-López et al., 2006; Lagdon et al., 2022) found that females were more likely to belong in the classes reflecting more severe abuse than males. Worthy of note, Carbone-López et al. (2006) assigned similar labels to classes found among women and men, yet the corresponding classes were frequently characterized by a different level of risk of experiencing IPV victimization. For example, men in the “systematic abuse” class had 100% risk of physical abuse, 1% risk of sexual assault, and 16% risk of stalking. Women in a class with the same label, in turn, had 100% risk of physical abuse, 23.8% risk of sexual assault, and 46.6% risk of stalking. Consequently, this attempt to retain labeling consistency across samples (which has also been identified in other studies) can obscure the true nature of IPV experienced by those samples and should be avoided in future studies. In addition, three studies specifically excluded non-heterosexual couples from their analyses (Ansara & Hindin, 2010; Clark, Cheong, et al., 2019; Gupta et al., 2018), and while other studies did not categorically exclude SGM individuals, only one study (Whitton et al., 2019) specifically examined how IPV patterns may be configured uniquely for this population. Combining these findings once again points to the fact that although IPV is not gender or population specific, it affects disproportionately more women than men (Ansara & Hindin, 2010; Carbone-López et al., 2006), and especially those from marginalized communities (Gupta et al., 2018; Whitton et al., 2019). Indeed, in marginalized communities, including lesbian–gay–bisexual–transgender–questioning–intersex and low-income communities, IPV is “embedded in a structural context in which individualized patterns of violence are mediated by legacies of structural oppression, discriminatory policies and lack of access to resources” (Ghanbarpour et al., 2018, p. 522). Therefore, appropriate support systems are critical to ensure that socioeconomic and demographic vulnerabilities do not increase the risk of IPV.

The study methodologies also varied considerably in terms of the range of IPV indicators they included, reflecting the discordance which surrounds the definition of IPV. Notably, one study included a measure of stalking (Carbone-López et al., 2006) and one study included measures of SGM-specific IPV and cyber dating abuse (Whitton et al., 2019). Discrepancies also arose regarding the inclusion of psychological abuse as some studies differentiated between psychological IPV and more specific components, such as coercive control (including such specific forms as reproductive coercion) and controlling behaviors (Leyton, 2020; McNaughton Reyes et al., 2019, 2021; Whitton et al., 2019; Willie et al., 2020), verbal abuse (Liu et al., 2018), jealousy (Ansara & Hindin, 2010; Lysova & Dim, 2020), and financial abuse (Ansara & Hindin, 2010). Additionally, two studies omitted measures of psychological abuse completely (Carbone-López et al., 2006; Gupta et al., 2018). Carbone-López et al. (2006)—the earliest study in the review—explained that measures of psychological abuse were omitted from their study to remain in line with the extant research of the time, which had not yet established a clear operational definition or empirical understanding of psychological abuse. Thus, while our understanding of psychological abuse has certainly improved, insights could be improved by including measures of psychological IPV more consistently. For example, Ansara and Hindin (2010) asked participants to report IPV from all relationships across their lifetime, however, where IPV occurred in more than one relationship, the researchers opted to only analyze relationships which included physical or sexual abuse, as psychological abuse was treated as less serious. Furthermore, Leyton (2020) investigated psychological IPV in current relationships but omitted it from investigations of past relationships. Research has repeatedly shown that psychological abuse is not only the most common form of IPV (Dokkedahl et al., 2019), but it can also have more harmful and longer-lasting negative outcomes than physical or sexual abuse (Jewkes, 2010). This further highlights the importance of including psychological abuse in IPV research and remedying the misconceptions that exist.

Another possible reason for inconsistent class solutions relates to the difference in time periods measured throughout the studies. Although the 12 months prior to study participation was the most common time period, other measurements ranged from the previous 6 months, previous 5 years, any time during the past relationship, any time during the current relationship, and across the whole lifetime. This variation is noteworthy as interpretation of the retrieved latent classes should be mindful of whether the classes represent IPV experiences at a single time point, the trajectory of victimization across one specific relationship, or the trajectory of abuse across a lifetime incorporating multiple abusive relationships. For example, while cessation of IPV is often observed in relationships where the abuse is less severe (Ansara & Hindin, 2010), it is also reported that psychological abusers may turn to physical abuse if their aims are not fulfilled (Tanha et al., 2010), meaning that if participants are asked to report only with regard to a single or short time period, researchers are unable to observe and understand factors which may lead to discontinuation of abuse, or conversely, the development of other forms of IPV. In addition, a common trajectory of psychological abuse resembles intense affection at the beginning of the relationship to obtain power and control—commonly referred to as “love bombing” (Duron et al., 2021). This means that if the relationship is new at the time of study participation, the abuse may not become apparent until later and the no/low abuse classes—which were consistently the most populous classes in studies which recruited community samples—could therefore be overrepresented. Cale et al. (2017) and McNaughton Reyes et al. (2019, 2021) were the only studies to specify a minimum relationship length as part of their eligibility criteria. Information regarding trajectories of how abuse develops and whether IPV evolves into different types of abuse can contribute toward the prevention of IPV worsening and the detection of risk factors related to re-victimization.

Studies also differed with regard to whether IPV was assessed specifically in relation to the victim’s current or previous partners. Abuse from former (rather than current) relationships is more commonly reported, especially in the case of severe abuse (Johnson et al., 2014), suggesting that victims may be more reluctant to report abuse when it remains ongoing. Ansara and Hindin (2010) found that the most severe pattern of violence was predominantly reported in relation to ex-partners. Furthermore, over 90% of victims reporting about their current partner were classified into the “no violence” group, whereas only 51.4% of women and 66.2% of men in the ex-partner sample reported experiencing no abuse. This finding may be a reflection of underreported incidents which may occur due to the victim hoping or believing that the abuse will end, believing that the abuse was their own fault or feeling sympathetic toward their abuser, being unable to provide honest responses while in the presence of their abuser, the victim fearing that they will not be able to support themselves or their children should the relationship end, or perceiving the seeking of help to be unacceptable as a social norm (Ansara & Hindin, 2010; Chadambuka, 2022; Podaná, 2021). Furthermore, Lysova and Dim (2020) found that male IPV victims are less likely to seek formal help when they are unemployed, less educated or have young children; possibly, the result of concerns that formal help would lead to the relationship ending or their children being taken away.

Finally, investigation of the relationship between latent classes and external criteria is crucial in establishing external validity of the retrieved classes and supports the choice of class solution by demonstrating that individuals are affected differently depending on class membership. This suggests that the effects of intervention and prevention methods will differ depending on the pattern of abuse suffered (Gulliver & Fanslow, 2015). Willie et al. (2020) were the only study not to include an analysis between latent classes and external criteria, while 10 out of the 14 studies examined IPV consequences and four investigated risk factors of IPV. In one study (Ansara & Hindin, 2010), the derived latent classes were assessed against violence-related characteristics (such as number of episodes of violence, partner drinking). Results consistently demonstrated an increase in negative outcomes as the severity of abuse worsened. Furthermore, the studies also showed that age, race, gender, sexuality, country of residence, relationship status, and socioeconomic status were all differentially associated with class membership (Liu et al., 2018; McNaughton Reyes et al., 2021; Podaná, 2021; Whitton et al., 2019). Importantly, the assessment of such relationships can be used to inform the development of prevention and intervention techniques and the administration of effective help to victims by establishing clear associations between risk factors, consequences, and specific IPV patterns. In particular, Lysova and Dim (2020) examined factors which influence help-seeking behaviors in men, which can be used to aid the improvement of males reporting their victimization experiences.

### Recommendations for Future Research

As illustrated above, there are numerous inconsistencies within the procedures and findings across the studies and further exploration of gaps in the literature are required. Recommendations for future research are listed below, including how to promote concord in future research and how to diversify and expand our knowledge of IPV experience.

1. A wide range of fit indices should be consulted when conducting LCA/LPA to ensure the optimal class solution is selected, and these indices should be reported to aid readers in determining the reliability of results. Considerations of parsimony and interpretability should also influence class enumeration.
2. When interpreting latent classes, comparing classes across studies and generalizing results to wider populations, particular information should be considered including whether the study uses a clinical or community sample, the time period which was studied, whether IPV was reported in relation to the current or former partner, and sociodemographic variables such as the gender of victim.
3. Some class labels, especially those referring to no/low IPV experiences, appear confusing because they are frequently not reflective of the actual risks of IPV victimization experienced by those group members. To systemize research in the area, it may be helpful if the research community agreed on the level of risk above which a class should not be labeled “no IPV” and “low IPV.” We propose that only groups with the level of risk on each IPV item included in the analysis that does not exceed 5% are labeled “no IPV” and those with risk that does not exceed 10% are labeled “low IPV.”
4. Researchers should aim to recognize the importance of including various indicators of psychological abuse in analyses and consider that psychological abuse does not automatically reflect a less severe IPV pattern, given the potential severity of the long-term consequences.
5. Future research should investigate IPV experiences within various marginalized populations.
6. In the current review, 11 studies utilized samples from Western countries. Future research should consider sampling from non-Western populations.

### Conclusions and Limitations

This systematic review provides a synthesized outline of 14 studies which use LCA to elucidate patterns of IPV victimization. The no/low violence pattern was consistently the most populated group within community samples, although some classes given this label recorded moderate levels of some IPV forms. Psychological abuse was the most inconsistently operationalized across studies, and researchers should strive to rectify this in future studies. Furthermore, although the proposed class solutions varied, external validity was established throughout all studies which included analyses between class membership and external criteria, suggesting that these differences may have arisen from methodological features such as the sample being studied, the time period and relationship specified in the study, and the range of IPV indicators being assessed. However, when interpreting the results presented in this review, certain limitations should be considered. First, only papers published in English were included, meaning that important findings published in other languages may have been omitted. This could also account for the under-representation of non-Western populations observed throughout these papers. Second, the use of exclusively peer-reviewed papers could be prone to publication bias (Table 2).

**Table 2.** Implications of the Review for Practice, Policy, and Research.

Practice	• Consider the plausibility of tailoring interventions to the individual based on the pattern of IPV experienced
	• Find ways to encourage victims to report ongoing IPV
Policy	• Develop a commonly agreed upon definition of IPV, using methods such as LCA/LPA to understand whether subtypes should be equally weighted
	• Results imply that marginalized populations are most likely to experience multiple IPV victimization. Prioritize development of interventions which focus on ameliorating the impact on victims from marginalized communities
Research	• Identify specific etiological factors associated with worse outcomes within typologies
	• Examine the impact of methodological features such as time period (e.g., last 12 months vs. ever) on IPV classes
	• Ensure that studies employing LCA/LPA assess external criteria to establish external validity of class solution
	• Examine why individuals are less likely to report ongoing IPV compared to historical IPV

IPV = intimate partner violence; LCA = latent class analysis; LPA = latent profile analysis.
